# Should biomedical research with great apes be restricted? A systematic review of reasons

**DOI:** 10.1186/s12910-021-00580-z

**Published:** 2021-02-16

**Authors:** Bernardo Aguilera, Javiera Perez Gomez, David DeGrazia

**Affiliations:** 1 Department of Bioethics, The Clinical Center, National Institutes of Health, Bethesda, USA; 2 Department of Bioethics and Medical Humanities, Faculty of Medicine, University of Chile, Santiago, Chile; 3 Department of Philosophy, Marquette University, Milwaukee, USA; 4 Department of Philosophy, George Washington University, Washington, USA; 5 Department of Bioethics, NIH Clinical Center, Bldg. 10, Room 1C118, Bethesda, MD 20892-115 USA

**Keywords:** Biomedical research, Great apes, Ethics, Systematic review, Animal experimentation

## Abstract

**Background:**

The use of great apes (GA) in invasive biomedical research is one of the most debated topics in animal ethics. GA are, thus far, the only animal group that has frequently been banned from invasive research; yet some believe that these bans could inaugurate a broader trend towards greater restrictions on the use of primates and other animals in research. Despite ongoing academic and policy debate on this issue, there is no comprehensive overview of the reasons advanced for or against restricting invasive research with GA. To address this gap, we conducted a systematic review of the reasons reported in the academic literature on this topic.

**Methods:**

Seven databases were searched for articles published in English. Two authors screened the titles, abstracts, and full texts of all articles. Two journals specialized in animal ethics, and the reference lists of included articles were subsequently also reviewed.

**Results:**

We included 60 articles, most of which were published between 2006 and 2016. Twenty-five articles argued for a total ban of GA research, 21 articles defended partial restrictions, and 14 articles argued against restrictions. Overall, we identified 110 reason types, 74 for, and 36 against, restricting GA research. Reasons were grouped into nine domains: moral standing, science, welfare, public and expert attitudes, retirement and conservation, respect and rights, financial costs, law and legal status, and longer-term consequences.

**Conclusion:**

Our review generated five main findings. First, there is a trend in the academic debate in favor of restricting GA research that parallels worldwide policy changes in the same direction. Second, in several domains (e.g., moral standing, and respect and rights), the reasons were rather one-sided in favor of restrictions. Third, some prominent domains (e.g., science and welfare) featured considerable engagement between opposing positions. Fourth, there is low diversity and independence among authors, including frequent potential conflicts of interests in articles defending a strong position (i.e., favoring a total ban or arguing against restrictions). Fifth, scholarly discussion was not the norm, as reflected in a high proportion of non-peer-reviewed articles and authors affiliated to non-academic institutions.

## Background

Historically, the debate over the use of animals in biomedical research has been divided between those who argue that animal research is necessary for medical progress and therefore justified, and those who favor restricting or even banning animal research. But even among proponents of animal research, there is growing concern regarding animal welfare. Indeed, many countries have introduced regulations aimed at improving the conditions under which animals are used in research. Perhaps the most notable development in the field of animal research regulation concerns the use of great apes (chimpanzees, bonobos, gorillas, and orangutans).^1^ In 2015, the (U.S.) National Institutes of Health joined the governments of Australia, New Zealand, Japan, and the European Union (E.U.) in banning or severely limiting experiments on chimpanzees [1].

Some commentators have suggested that the turn to ban invasive biomedical research with great apes (hereafter, GA) could represent the beginning of a more general trend towards increasing restrictions on the use of primates and other animals in research [2–4].^2^ Recent developments seem to confirm this hypothesis. For example, in 2018, a U.S. Senator introduced legislation that would severely restrict the use of non-human primates in biomedical research, and in 2020 the U.S. National Academies of Sciences, Engineering, and Medicine published a report that recommended more stringent conditions on the use of dogs in research funded by the U.S. Department of Veterans Affairs (NASEM) [5, 6].

Since GA constitute the only animal group that has consistently been banned from invasive research in many countries, the reasons given for and against such a ban—or other restrictions—can provide a basis for judging whether invasive research on other animal species should be restricted. However, these reasons are scattered in the literature and often come from sources swayed by one or the other side of the debate. To address these concerns, we conducted a systematic review of the reasons advanced for and against restricting research with GA. Our review is a valuable contribution to the debate over whether GA should be used in invasive biomedical research in three main respects. First, by mapping the ethical debate on this issue, this review identifies argumentative patterns, gaps, and underrepresented concerns, thereby revealing alternative directions for advancing the debate. Second, our review can provide a basis for judging the adequacy of reasons given for and against extending research restrictions to other animal species or groups. Finally, it can help policymakers and regulators make fully informed and minimally biased decisions concerning the regulation of GA research.

## Methods

We performed a systematic review of reasons, a type of review that provides a comprehensive and systematic account of the reasons given in the literature in connection with an ethical issue, and that is primarily descriptive rather than evaluative [7]. We followed the PRISMA Statement and checklist in formulating this review [8].

### Search strategy

A medical librarian searched seven bibliographic databases covering the health sciences (PubMed, Global Health), life sciences (Web of Science: Core Collection, Web of Science: BIOSIS Citation Index, Web of Science: Zoological Record) and ethics (EthxWeb, PhilPapers), using a combination of keywords and controlled vocabularies. We limited the searches to English language and did not limit by publication date. The searches were completed in July 2019 and updated in July 2020. EndNote X9 (Clarivate Analytics) was used to collect the citations and identify duplicates. The final search strategies for each database are listed in Additional file 1: Table 1.

We also hand-searched the electronic table of contents of two journals specialized in animal ethics (*Between the Species* and *Journal of Animal Ethics*) to identify additional relevant articles. Finally, we screened the reference lists of included articles for additional articles to consider.

### Article selection and inclusion criteria

The first and second authors (BA, JPG) independently screened the retrieved articles using pre-established inclusion or exclusion criteria in two steps: first by reviewing titles and abstracts, and then by reviewing the full texts of those included in the first step. Google Sheets was used for the article screening process. The two authors jointly resolved disagreements over the eligibility of publications, and any remaining disagreements were resolved through discussion with the third author (DD).

We included a publication if all of the following criteria were met:

1. It specifically discussed the ethics or regulation of research with GA (or some of the GA species).
2. It addressed reasons why the use of GA for research should or should not be restricted or banned.
3. It considered research that was invasive (that is, potentially harmful and not primarily aimed to benefit the individual or the species to which it belongs).
4. It was an article (understood broadly, to include various types of journal writings such as commentaries and letters) published in English in an academic journal.

Inclusion criteria (1) and (2) excluded purely empirical veterinary or biomedical research and articles not endorsing reasons for or against restricting GA research (e.g., news articles or purely descriptive reviews of the debate). Articles discussing the regulation of animal research more generally were eligible only if they offered reasons that explicitly applied to GA research (e.g., in claiming that there is a stronger case for restricting GA research than for research involving other types of animals). Articles focusing on the ethics of GA research were included only if they addressed research regulation. We employed criterion (3) to exclude articles that addressed noninvasive forms of GA research only (e.g., purely observational studies), as well as research intended to benefit GA exclusively. Hereafter, unless otherwise specified, references to “GA research” should be understood to refer only to *invasive, nontherapeutic* GA *biomedical* research.

### Data extraction and analysis

The first and second authors (BA, JPG) independently reviewed the full texts of the included articles and extracted reasons for or against restriction of GA research. Google Sheets was used for the data extraction and coding. We counted a claim as a reason when it was advanced independently by the author; we did not count mentions of others’ claims as a reason unless they were actively endorsed by the author. We assigned mentions of reasons to specific categories (reason types) and grouped them into nine broader domains. This was carried out by highlighting and labeling passages of reasons, and grouping them into categories, using inductive and deductive content analysis processes [9, 10]. Depending on the overall position taken by the authors, we categorized articles favoring restrictions into *total ban* or *partial restrictions*, and those opposing restrictions into *against restrictions*. For comprehensiveness, we categorized articles into *against restrictions* even if they adopted this position implicitly: by taking a favorable attitude towards GA research, without specifically mentioning restrictions. In general, with the exception of some articles supporting partial restrictions that contained a mix of reasons for either position, reasons within articles were either strictly for or strictly against GA research.

Within some domains, we classified reason types according to subdomains. When a reason type seemed to apply to more than one domain, we classified the reason under the domain we considered most appropriate based both on its content and on how informative it would be to the reader. The first and second authors (BA, JPG) performed this process in close collaboration, through frequent meetings to revise the data extraction and analysis in order to concur on the coding of the reasons. For remaining disagreements, the third author (DD) participated in discussions until consensus was reached on how to resolve the disagreement in coding.

We classified journals using the All Science Journal Classification (ASJC) scheme in physical sciences, health sciences, life sciences, and social sciences & humanities (SS&H) [11]^3^ When the journal fit into more than one field, or the journal was not listed in ASJC (which occurred in three cases), we used the journal's webpage description to determine the journal’s classification. One journal (*Bulletin of the National Society for Medical Research*) is no longer active so we based its classification on its title.

We identified an article as posing a potential conflict of interest when the journal in which the article was published or any of the articles’ authors were affiliated with or sponsored by an institution that, according to its webpage (e.g., a mission or vision statement), takes a position in favor of or against the use of GA or animals more generally in biomedical research (Additional file 1: Table 2). We developed this method as none of the articles in our review disclosed conflicts of interest as indicated by a conflict of interest statement in the article.

## Results

### Article characteristics

**Fig. 1:**
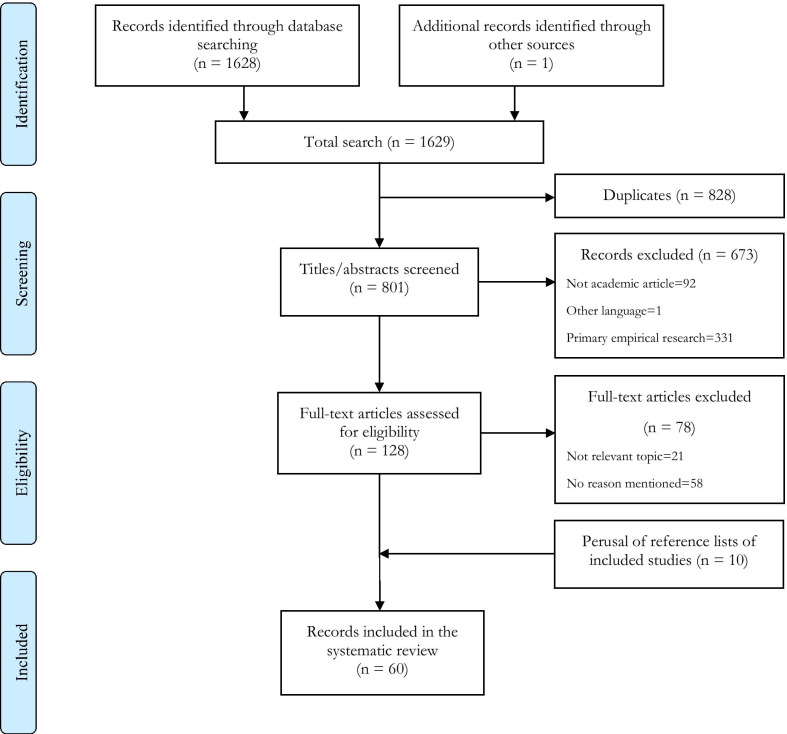
PRISMA flow chart of the selection process

**Fig. 2:**
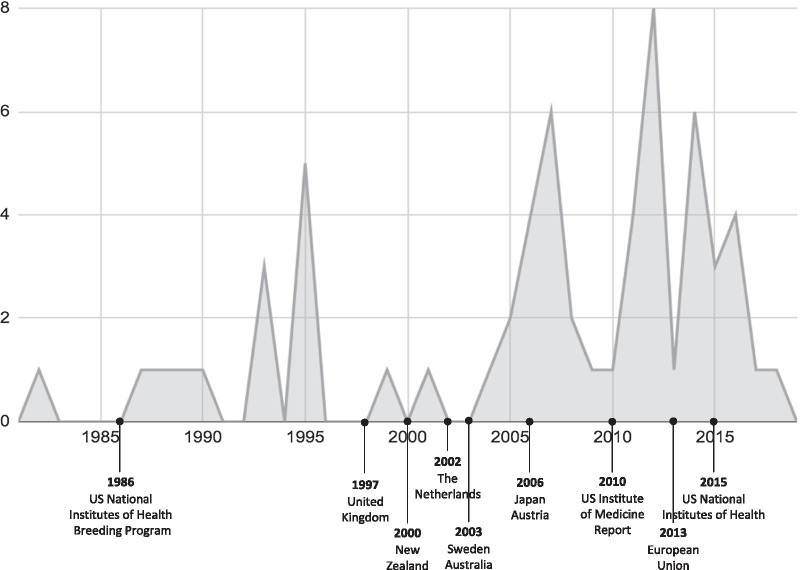
Number of publications included in this systematic review between 1980 and 2020, in relation to relevant historical events and names of countries/institutions in which great ape research has been severely restricted or banned

The database searches yielded 801 unique records. After title and abstract screening, full text screening, and perusing reference lists, 60 articles were included for data extraction and analysis (see Fig. 1; all included publications are listed in Table 1). The dates of publication ranged from 1982 to 2018, but 40 (67%) articles were published between 2006 and 2016 (see Fig. 2). Nearly half of the articles (42%) in our final list were types of opinion pieces (e.g., commentaries, letters, and editorials) (see Table 2). All articles discussed the research use of chimpanzees, which are the GA species that has historically been the most frequently used in invasive biomedical research. Other GA species were also mentioned in 18 articles, but often in passing or to provide some context for the discussion of chimpanzee research.

**Table 1:** Included and analyzed publications in alphabetical order

[12]	Altevogt BM, Pankevich DE, Pope AM, Kahn JP. Guiding limited use of chimpanzees in research. Science. 2012;335:41–2
[13]	Bailey J. An examination of chimpanzee use in human cancer research. Altern Lab Anim. 2009;37:399–416
[14]	Bailey J. Biomedical research involving chimpanzees. Altern Lab Anim. 2011;39:413–4
[15]	Bailey J. Lessons from chimpanzee-based research on human disease: the implications of genetic differences. Altern Lab Anim. 2011;39:527–40
[2]	Balls M. Chimpanzee medical experiments: Moral, legal and scientific concerns. Altern Lab Anim. 1995;23:607–14
[16]	Balls M. Primates in medical research: The plot thickens. Altern Lab Anim. 2006;34:271–2
[17]	Balls M. Time for real action on chimpanzees and other hominids. Altern Lab Anim. 2007;35:191–5
[18]	Beauchamp TL, Ferdowsian HR, Gluck JP. Where are we in the justification of research involving chimpanzees? Kennedy Inst Ethics J. 2012;22(3):211–42
[19]	Beauchamp TL, Wobber V. Autonomy in chimpanzees. Theor Med Bioeth. 2014;35:117–32
[20]	Bennett AJ. New era for chimpanzee research: Broad implications of chimpanzee research decisions. Dev Psychobiol. 2015; 10.1002/dev.21294
[21]	Bennett AJ, Panicker S. Broader impacts: International implications and integrative ethical consideration of policy decisions about US chimpanzee research. Am J Primatol. 2016; 10.1002/ajp.22582
[22]	Bloomsmith MA, Schapiro SJ, Strobert EA. Preparing chimpanzees for laboratory research. ILAR J. 2006; 47(4):316–25
[23]	Bradshaw GA, Capaldo T, Lindner L, Grow G. Building an inner sanctuary: Complex PTSD in chimpanzees. J Trauma Dissociation. 2008;9:8–34
[24]	Capaldo T, Peppercorn M. A review of autopsy reports on chimpanzees in or from US laboratories. Altern Lab Anim. 2012;40:259–69
[25]	Cavalieri P. Ethics, animals and the nonhuman great apes. J Biosci. 2006;31(5):509–12
[3]	Cavalieri P, Singer P. The great ape project: Premises and implications. Altern Lab Anim. 1995;23:626–31
[26]	Conlee KM. Chimpanzees in research and testing worldwide: Overview, oversight, and applicable laws. AATEX 14 (Special Issue). 207;14:111–18
[27]	Conlee KM, Hoffeld EH, Stephens ML. A demographic analysis of primate research in the United States. Altern Lab Anim. 2004;32 Suppl 1:315–22
[28]	de Waal FB. Research chimpanzees may get a break. PLoS Biol. 2012;10(3):1–4
[29]	DeGrazia D. Human-animal chimeras: Human dignity, moral status, and species prejudice. Metaphilosophy. 2007;38:309–29; https://doi.org/10.1111/j.1467-9973.2007.00476.x
[30]	DeGrazia D. Nonhuman primates, human need, and ethical constraints. Hastings Cent Rep. 2016;46(4):27–28; https://doi.org/10.1002/hast.601
[31]	Eichberg JW, Speck JT. Establishment of a chimpanzee retirement fund: Maintenance after experimentation. J. of Med. Primatol. 1988;17:71–6
[32]	Fenton A. Can a chimp say "no"? Reenvisioning chimpanzee dissent in harmful research. Camb Q Healthc Ethics. 2014;23:130–9
[33]	Fenton A. On the need to redress an inadequacy in animal welfare science: Toward an internally coherent framework. Biol Philos. 2012;27:73–93; https://doi.org/10.1007/s10539-011-9291-1
[34]	Fultz PN. Nonhuman primate models for AIDS. Clin Infect Dis. 1993;17 Suppl 1:S230–35
[35]	Gagneux P, Moore JJ, Varki A. The ethics of research on great apes. Nature. 2005;437:27–9
[36]	Goodall J. Ending research on non-human primates. ALTEX. 2005;22:14–8
[37]	Goodall J. Why is it unethical to use chimpanzees in the laboratory. Altern Lab Anim. 1995;23:615–20
[38]	Great ape debate. Nature. 2011;474:252; https://doi.org/10.1038/474252a
[39]	Gruen L. The end of chimpanzee research. Hastings Cent Rep. 2016; https://doi.org/10.1002/hast.604
[40]	Jacobs L. The use and the care of the chimpanzee. Bull Natl Soc Med Res. 1982;33(2):1–2
[41]	Johnson K. The misuse of chimpanzees in biomedical experiments. Altern Lab Anim. 1995;23:648–51
[42]	Johnson J, Barnard ND. Chimpanzees as vulnerable subjects in research. Theor Med Bioeth. 2014;35:133–141; https://doi.org/10.1007/s11017-014-9286-4
[43]	Jones RC, Greek R. A review of the Institute of Medicine's analysis of using chimpanzees in biomedical research. Sci Eng Ethics. 2014;20:481–504; https://doi.org/10.1007/s11948-013-9442-7
[44]	Kahn J. Lessons learned: Challenges in applying current constraints on research on chimpanzees to other animals. Theor Med Bioeth. 2014;35:97–114; https://doi.org/10.1007/s11017-014-9284-6
[45]	Kantin H, Wendler D. Is there a role for assent or dissent in animal research? Camb Q Healthc Ethics. 2015;24:459–72; https://doi.org/10.1017/S0963180115000110
[46]	Knight A. Assessing the necessity of chimpanzee experimentation. ALTEX. 2012;29:93–2
[47]	Knight A. The beginning of the end for chimpanzee experiments? Philos Ethics Humanit Med. 2008; 10.1186/1747-5341-3-16
[48]	Knight A. The poor contribution of chimpanzee experiments to biomedical progress. J Appl Anim Welf Sci. 2007;10(4):281–308
[49]	Kraska, K. Are we justified in conducting invasive research on captive apes for their wild counterparts? Soc Anim. 2018;26:598–615
[50]	LaManna JC. Animal models: Ads against chimp research criticized. Nature. 2012;483:275
[51]	Lanford RE, Walker CM, Lemon SM. The chimpanzee model of viral hepatitis: Advances in understanding the immune response and treatment of viral hepatitis. ILAR J. 2017;58(2):172–89
[52]	Latzman RD, Hopkins WD. Letter to the editor: Avoiding a lost opportunity for psychological medicine: importance of chimpanzee research to the National Institutes of Health portfolio. Psychol Med. 2016;46:2445–7
[53]	Lopresti-Goodman SM, Bezner J, Ritter C. Psychological distress in chimpanzees rescued from laboratories. J Trauma Dissociation. 2015;16(4):349–66; https://doi.org/10.1080/15299732.2014.1003673
[54]	McKellips P. The slippery slopes with Tommy, Kiko, Hercules, Leo and Duke. Lab Anim (NY). 2014;43(2):69
[55]	Participants, Primate Workshop 1987, Washington DC. Recommendations to USDA on improving conditions of psychological well-being for captive chimpanzees. Altern Lab Anim. 1988;15:255–60
[56]	Prince AM. Is the conduct of medical research on chimpanzees compatible with their rights as a near-human species? Between Species. 1993
[57]	Prince AM, Allan J, Andrus L, Brotman B, Eichber J, Fouts R, et al. Virulent HIV strains, chimpanzees, and trial vaccines. Science. 1999;283(5405):1117
[58]	Prince AM, Brotman B, Garnham B, Hannah AC. Enrichment, rehabilitation, and release of chimpanzees used in biomedical research: procedures used at Vilab II, the New York Blood Center's Laboratory in Liberia, West Africa. Lab Animal. 1990;19:29–37
[59]	Prince AM, Goodall J, Brotman B, Dienske H, Schellekens H, Eichberg JW. Appropriate conditions for maintenance of chimpanzees in studies with blood-borne viruses: An epidemiologic and psychosocial perspective. J. Med. Primatol. 1989;18:27–42
[60]	Reynolds V. Moral issues in relation to chimpanzees in gield studies and experiments. Altern Lab Anim. 1995; https://doi.org/10.1177/026119299502300512
[61]	Rowan AN. The uncertain future of research chimpanzees. Science. 2007;315:1493
[62]	Rowan A, Conlee K, Bettauer R. End invasive chimp research now. Nature. 2011;475:296
[63]	Taylor R. A step at a time: New Zealand's progress toward hominid rights. Animal Law. 2001;7(35):35–43
[64]	Thew M, Bailey J, Balls M, Hudson M. The ban on the use of chimpanzees in biomedical research and testing in the UK should be made permanent and legally binding. Altern Lab Anim. 2012;40:3–8
[65]	Van Akker R, Balls M, Eichberg JW, Goodall J, Heeney JL, Osterhaus A, et al. Chimpanzees in AIDS research – a biomedical and bioethical perspective. J. Med. Primatol. 1994;23:49–51
[66]	VandeBerg JL. Reclassification of captive chimpanzees as endangered would cost lives. Journal of Medical Primatology. 2013;42:225–8; https://doi.org/10.1111/jmp.12074
[67]	VandeBerg S. A unique biomedical resource at risk. Nature. 2005;437:30–2
[68]	Varki A. Fate of 'retired' research chimps. Nature. 2010;467:1047
[69]	Wise SM. The entitlement of chimpanzees to the common law writs of habeas corpus and de homine replegiando. Golden Gate University Law Review. 2007;37(2):219–80

**Table 2:** Characteristics of publications included in this systematic review

Publication characteristics	*N*	Percentage
*Affiliation of corresponding author*^a^		
Private Institution or Foundation	33	52
Public University	12	19
Private University	8	13
Governmental Organization	5	8
None	5	8
*Type of journal*		
Social Sciences & Humanities	16	27
Health Sciences	27	45
Life Sciences	17	28
*Type of publication*		
Original article	32	53
Commentary	14	23
Letter	8	13
Editorial	3	5
Conference proceedings	2	3
Workshop proceedings	1	2

^a^The numbers add up to more than the total number of 60, as some authors had more than one affiliation

Most authors were affiliated with an entity based in the U.S. (66%) or the U.K. (20%). Most U.K.-based authors favored a total ban (92%), whereas U.S.-based authors were almost evenly distributed between advocating for a total ban (31%), for partial restrictions (38%), or against restrictions (31%). The remaining authors were based in Italy (2), The Netherlands (2), Australia (1) and New Zealand (1). Institution-wise, 52% of corresponding authors were affiliated to a private institution or foundation, 19% to a public university, 13% to a private university, and 8% to a governmental organization.

In general, most articles were published in scientific journals (health sciences 45%, life sciences 28%) while 27% were in social sciences & humanities (SS&H) journals. However, some domains departed significantly from this distribution, as indicated in each relevant domain below. Finally, potential conflicts of interests were common, especially in articles defending strong positions: 84% in articles defending a  total ban, 23% in articles favoring partial restrictions, and 50% in articles against restrictions.^4^

### Positions taken by the authors

With respect to the overall positions taken by the authors of the included articles, 25 (42%) articles argued for a total ban of GA research, 21 (35%) articles defended partial restrictions, and 14 (23%) articles argued against restrictions. Of those that defended partial restrictions, 13 (22%) argued for restrictions consistent with protections for certain human subjects—in particular, those who cannot provide informed consent or are designated as vulnerable (e.g., infants)—while 8 (13%) argued for less stringent protections. Despite the range of views identified, authors rarely cited other articles defending an opposing view: while 22 (37%) articles cited other articles approvingly (i.e., to support a proposed viewpoint) only 2 (3%) articles cited other articles to engage with an opposing viewpoint.

### Reasons for and against restricting invasive research with GA

We identified a total of 110 reasons: 74 in favor of and 36 against restricting GA research. These reasons were mentioned a total of 315 times of which 238 were in favor of and 77 against restrictions. We categorized these reasons into the following nine domains, listed in descending order of frequency: (1) Moral Standing, (2) Science, (3) Welfare, (4) Public and Expert Attitudes, (5) Conservation and Retirement, (6) Respect and Rights, (7) Financial Costs, (8) Law and Legal Status, and (9) Longer-term Consequences. Table 3 includes a detailed account of the reasons in each domain and their frequency.

**Table 3:** Reasons for and against restricting invasive research with great apes (GA)

Domain	Position	Subdomain and reasons	*N*	References
*Moral standing*			104	
		*Similarity to humans*	60	
	Pro	GA possess certain cognitive and behavioral capacities similar to humans, and thus deserve special protections	15	[2, 3, 17, 18, 25, 27, 28, 30, 32, 36, 37, 39, 55, 59, 60]
		GA and humans have a similar evolutionary origin, so GA deserve special protections	10	[3, 18, 25, 37, 39, 55, 59, 60, 63, 69]
		GA possess certain conscious experiences similar to humans, and thus deserve special protections	9	[2, 17, 18, 28, 32, 37, 39, 55, 59]
		Like humans, GA exhibit moral behavior, and thus deserve special protections	8	[2, 18, 28, 36, 37, 39, 47, 55]
		GA are greatly anatomically and/or physiologically similar to humans, and thus deserve special protections	6	[2, 18, 27, 32, 37, 55]
		There is great genetic similarity between GA and humans, so GA deserve special protections	5	[2, 35, 37, 55, 69]
		Like humans, GA have a long period of childhood dependency on the mother, so they deserve special protections	3	[36, 37, 59]
		GA are very similar to humans (unspecified), and thus deserve special protections	2	[3, 65]
		GA possess cognitive capacities similar to those of cognitively disabled humans, and thus deserve special protections	1	[60]
	Con	GA seem very similar to us, but this need not entail special protections since it may be the result of training or mimicking	1	[40]
		*Cognitive and consciousness-related capacities*	32	
	Pro	GA can have complex conscious experiences, so they deserve special protections	19	[3, 13–15, 18, 23, 25–27, 33, 35, 39, 41, 42, 47, 48, 53, 64, 69]
		GA have certain sophisticated cognitive capacities so they deserve special protections	13	[3, 16, 25–27, 33, 35, 37, 41, 46, 47, 63, 69]
		*Double standards*	8	
	Pro	Treating GA with less consideration than humans, without good reason, is speciesist	7	[18, 29, 32, 33, 42, 49, 63]
		Treating GA with less consideration than humans is inhumane	1	[47]
		*Vulnerability and dependency*	4	
	Pro	Captive GA can be considered vulnerable subjects, and thus deserve special protections	3	[18, 42, 49]
		Captive GA are in a special relation of dependency on humans, and thus deserve special protections	1	[49]
*Science*			89	
		*Scientific and medical value*	56	
	Pro	Current GA research has low medical value	12	[2, 13–15, 35, 41, 43, 46–48, 61, 64]
		GA research lacks significant scientific value (unspecified)	6	[13, 17, 27, 41, 43, 48]
		The medical value of past GA research need not predict the medical value of future GA research	2	[44, 62]
		Past GA research has been falsely credited as having high medical value	2	[15, 43]
		Even if the need of GA to combat an emerging diseases were justified, their use would not be possible for logistical and economic reasons	1	[64]
		The supposed need of GA research to combat emerging diseases is unjustified	1	[61]
		GA have not been key to combating emerging diseases	1	[26]
	Con	Current GA research has high medical value	9	[31, 34, 38, 50, 51, 56, 58, 59, 67]
		Past GA research has had high medical value	6	[31, 38, 51, 59, 66, 67]
		GA may be needed to combat future emerging diseases (e.g. Ebola)	4	[12, 50, 66, 67]
		Past GA research has had high scientific value	3	[12, 66, 67]
		Abandoning GA as research models may slow down medical discovery	2	[51, 66]
		Current GA research has high scientific value	1	[67]
		GA research is essential for reducing risks to human research subjects	1	[67]
		The medical value of past GA research is a good predictor of the medical value of future GA research	1	[67]
		The medical value of GA research may be higher than it seems, since some GA research supplied to regulatory agencies is never published	1	[67]
		GA research-based medical progress will become increasingly apparent with time	1	[51]
		GA research may become (even more) medically valuable as a result of new technologies	1	[67]
		Restricting GA research could cost human lives	1	[66]
		*Existence of alternative methods*	27	
	Pro	GA research is unnecessary (unspecified)	7	[12, 14, 24, 28, 37, 41, 60]
		Alternative, ethical methods (e.g., other animals or non-animal models) exist	6	[2, 15, 28, 41, 44, 62]
		Restricting GA research might drive scientists to develop alternative research methods	1	[26]
	Con	No alternative, ethical methods exist	8	[22, 31, 38, 51, 56, 59, 66, 67]
		GA research is necessary (unspecified)	3	[50, 58, 65]
		Major medical advances would not have been possible with alternative methods	2	[12, 67,]
		*Reliability of methods*	6	
	Pro	The methodology of current GA research is questionable (unspecified)	1	[41]
		GA used in labs often have multiple diseases and so are inappropriate research models, scientifically and ethically	1	[24]
		The stress that GA face in laboratory life can produce misleading research results	1	[14]
		The apparent genetic similarity between GA and humans need not entail that GA are appropriate research models	1	[15]
		GA have proved to be poor research models, so investing resources in them may hinder the advancement of medicine	1	[14]
	Con	Given the phylogenetic continuity between GA and humans, GA are good animal models for studying human diseases	1	[31]
*Welfare*			32	
	Pro	GA care and housing requirements are virtually impossible to meet	5	[2, 17, 26, 47, 61]
		The conditions of captive GA are appalling	4	[3, 37, 53, 64]
		GA care and housing requirements are not actually met	2	[37, 64]
		The conditions of captive GA can cause GA psychological harms	2	[26, 53]
		GA care and housing requirements are particularly high (unspecified)	1	[64]
		GA research sometimes significantly harms GA (unspecified)	1	[63]
		GA research sometimes significantly harms GA physically	1	[26]
		GA research sometimes significantly harms GA psychologically	1	[26]
		Since GA are long-lived, they are used for multiple protocols, which results in increased suffering	1	[64]
		Since GA are long-lived, they can be kept in laboratories for decades, which is unethical	1	[26]
		Captivity deprives GA of social learning, which is required for normal development	1	[55]
		The benefits of GA research do not outweigh the harms it causes GA	1	[64]
		Although there is great uncertainty regarding the nature and magnitude of GA suffering, we should assume that suffering may occur	1	[47]
	Con	GA care and housing requirements can actually be met	3	[40, 56, 59]
		GA research can be carried out without significantly harming GA	2	[22, 66]
		GA are better off in research facilities (e.g., in terms of life-expectancy or wellbeing) than in the wild	2	[56, 66]
		GA care in research facilities is adequate	1	[66]
		GA research is necessary for improving GA welfare	1	[21]
		Captive GA that are abandoned by their owners are better off in research facilities than in the wild since there are no available sanctuaries to keep them	1	[56]
*Public and expert attitudes*			24	
	Pro	Many other (developed) countries have already restricted GA research	12	[13, 15, 24, 26, 27, 30, 39, 46, 47, 53, 61, 64]
		There is opposition for GA use in research	8	[14, 15, 26, 28, 33, 39, 46, 64]
		Many pharmaceutical companies and private laboratories have already ended GA use	1	[14]
		Expert support for invasive GA research has declined	1	[61]
		GA scientists now share concern about GA research	1	[26]
		GA research sometimes requires euthanizing GA, but euthanizing GA is widely condemned	1	[57]
*Conservation and retirement*			20	
	Pro	Supplying GA for research has led to a decline of wild populations and the threat of extinction	2	[2, 60]
		GA are endangered species (unspecified)	2	[26, 64]
		Optimal GA retirement should be to return them to the wild, but this is not feasible	1	[35]
		Appeals to conservation do not justify breeding GA in captivity for research	1	[47]
	Con	Conservation efforts could benefit from GA research	4	[20, 21, 35, 66]
		GA could be cared for after research by moving them to near-wild conditions	3	[56, 58, 59]
		GA research could improve the welfare and protection of GA as a species	2	[20, 21]
		Enough captive GA are already available for research	2	[56, 65]
		Breeding captive GA for research could ensure the survival of the species	1	[66]
		GA could be cared for after research by moving them to other research facilities	1	[40]
		GA could be cared for after research by moving them to indoor/outdoor facilities	1	[31]
*Respect and rights*			15	
	Pro	GA are capable of assenting/dissenting (like children)	5	[19, 32, 33, 42, 45]
		GA can be considered subjects with diminished or no capacity for informed consent	3	[3, 48, 68]
		GA possess enough cognitive capacities to be considered persons	3	[3, 25, 49]
		GA possess enough cognitive capacities to be considered near-persons or person-like	2	[29, 30]
		Given that GA have the same capacities we cite for humans having the moral right to life, freedom, and welfare, GA should also be conceived as having these rights	1	[25]
		Given that GA have the capacities that may form the foundation of personhood, they have a moral right against our intentional infliction of harm	1	[49]
*Financial costs*			13	
	Pro	Required GA care and housing costs are too high to be cost-effective	3	[2, 47, 61]
		Required GA care and housing costs are particularly high	2	[2, 35]
		The financial costs of GA research are particularly high	2	[14, 46]
		The benefits of GA research do not outweigh the financial costs	1	[13]
		Given that GA are long-lived, the costs of GA care and housing after research is particularly high	1	[60]
		Funding for GA research continues to decrease, while the costs of GA research continues to increase	1	[26]
	Con	Many experiments could be carried out with just a small population of GA	1	[67]
		Given that GA are long-lived, the costs of GA care and housing after research is high but manageable	1	[31]
		Restricting GA research could increase medicine costs	1	[66]
*Law and legal status*			11	
	Pro	Some laws and policies already restrict the use of GA for research	3	[15, 30, 59]
		Given their cognitive capacities, GA should be granted legal personhood	2	[63, 69]
		GA should be granted the legal right to liberty	1	[69]
		GA should be granted the legal right not to be subjected to experiments that are not in their best interests	1	[63]
		GA should be granted the legal right to personal security	1	[63]
		GA should be granted the legal right to life	1	[63]
	Con	Laws and policies protecting GA vary in terms of strictness depending on setting (research, zoos, or private homes)	1	[21]
		Granting legal personhood to GA is a slippery slope into granting legal personhood to other animals	1	[54]
*Longer-term consequences*			7	
	Pro	Restricting GA research is instrumental for restricting research on other animal species	3	[2, 17, 33]
		Restricting GA research is an important first step away from speciesism against GA	1	[63]
		Restricting invasive GA research need not have a negative impact on non-invasive GA research	1	[16]
	Con	Restricting GA research will have a negative impact on non-invasive GA research	2	[21, 52]
Total			315	

#### Moral standing

This domain comprises reasons that appeal either directly or indirectly to the moral standing of GA as grounds for giving them special protections. Reasons in this domain were the most frequently mentioned in the literature and were overwhelmingly in favor of restricting GA research (103 mentions in favor and 1 against). Authors offered various grounds for granting GA special protections, which we further categorized in descending order of frequency into four subdomains: (a) Similarity to Humans, (b) Cognitive and Consciousness-related Capacities, (c) Double Standards, and (d) Vulnerability.

##### Similarity to humans

Many authors appealed to perceived similarities between GA and humans as grounds for giving GA special protections. We interpreted this reasoning as an argument from analogy, inferring a similar moral standing between humans and GA given that they share relevant similarities. Reasons in this domain figured predominantly in health sciences journals. Furthermore, reasons in this domain were largely used in favor of restricting GA research (59 mentions in favor and 1 against). Some of the most frequently cited reasons conceived of the relevant similarities in terms of cognitive and consciousness-related capacities. Authors supported these reasons by invoking several more specific capacities (see Table 4). Other reasons for granting GA special protections on grounds of their similarity to humans appealed to genetic, evolutionary, developmental, and behavioral characteristics. It is worth noting that the only reason against restrictions in this subdomain was that while GA may *seem* very similar to humans with respect to certain behaviors, this seeming similarity may be the result of training or mimicry.

**Table 4:** Cognitive and consciousness-related capacities used as grounds for granting great apes special protections

Capacity for	Mentions
Social interaction	14
Complex or sophisticated emotions	13
Rational thought	12
Intense suffering	11
Self-awareness	11
Post-traumatic disorders	9
Sophisticated, human-like capacities (unspecified)	8
Tool use	7
Gestural communication	7
Language acquisition	6
Culture development	5
Imitative learning	5
Symbolic representation	4
Abstract thought	4
Prospective thinking or planning	3
Working or episodic memory	3
Mind reading	3
Complex calculation	2
Social deprivation	2
Sense of humor	2
Sophisticated, human-like capacities in infancy	2
Personality development	1
Social cooperation	1

##### Cognitive and consciousness-related capacities

Reasons in this subdomain appeal to granting GA special protections based on their cognitive and consciousness-related capacities, regardless of their similarity to humans. These reasons were raised most frequently in SS&H journals and were solely used in favor of restrictions. Drawing on the literature on moral standing, we interpreted these reasons as giving moral weight to the possession of certain capacities. Since these were often put forward as independent claims, we distinguished between reasons appealing to consciousness-related capacities and reasons referring to other sophisticated cognitive capacities attributed to GA. As in the previous subdomain, authors sometimes mentioned more specific capacities, which we list in Table 4.

##### Double standards

Reasons in this subdomain make implicit or explicit appeals to the moral standing of GA to argue that not giving GA special protections would amount to unjustifiably giving less moral consideration to GA than to humans. Most authors argued that this would be speciesist, while one author argued that it would be inhumane. All of these reasons were published in SS&H journals.

##### Vulnerability and dependency

This subdomain comprises reasons that appealed to the vulnerable status of GA as research subjects to argue for restrictions on GA research. We categorized reasons about vulnerability and dependency in the Moral Standing domain because these notions often arise in virtue of the relationship between human and nonhuman animals, and such relationships are sometimes taken to be relevant grounds for moral consideration [70, 71]. These reasons, which appeared exclusively in SS&H journals, evinced two main senses of vulnerability: the vulnerability intrinsic to GA as biological beings (e.g., vulnerability to disease, illness, or psychological harm), and the situational vulnerability of being used as research subjects (e.g., the risk of exploitation and increased harm, especially given captive GA’s status of dependency on researchers).

#### Science

Reasons related to science figured prominently in the debate and were used almost equally to argue for and against restricting GA research (44 mentions in favor and 45 against). Reasons in favor were much more common in health sciences journals while reasons against were mostly mentioned in life sciences journals. We subcategorized these reasons, in order of frequency, into three subdomains: (a) Scientific and Medical Value, (b) Existence of Alternative Methods, and (c) Reliability of Methods.

##### Scientific and medical value

This subdomain focuses on the value of invasive GA research for scientific discovery (what we call “scientific value”) as well as for improving health and well-being (what we call the “medical value” of GA research). This is the only subdomain containing more reasons against than reasons in favor of restrictions.

The main dispute within this subdomain concerns the medical value of GA research. Those against restrictions appealed to a putative past and the current medical value of GA research (particularly in relation to HIV and hepatitis viruses) or cautioned that abandoning GA as research models may slow down medical discovery. On the other side, reasons disputed the medical value of previous and current GA research. Similar reasons for and against restrictions were presented on the scientific value of GA experiments, concerning areas such as genomics and behavioral research.

Another point of contention in this subdomain relates to the potential role of GA research in combating future emerging diseases such as Ebola. Authors arguing against restrictions stressed the importance of keeping chimpanzee resources available for that purpose. In favor of restrictions, authors contended that the supposed need of GA to combat emerging diseases is not justified, and that even if it were, the use of GA would not be possible for logistical and economic reasons.

##### Existence of alternative methods

Several authors appealed to the (non)existence of alternative methods to argue for or against GA research. Some disagreed about the *necessity* of GA research, a term used to indicate that there are no acceptable alternatives for highly valuable biomedical research. Others debated whether alternative research methods were available. Authors favoring restrictions contended that alternatives (e.g., other animals, non-animal models, or human volunteers) do exist or that such restrictions may drive scientists to develop alternative methods (e.g., cell lines that can be infected with human viruses). Against restrictions, authors primarily argued that sufficient and appropriate alternative methods are not available (e.g., because chimpanzees are the only non-human animal model that can be infected successfully with certain viruses).

##### Reliability of methods

Reasons in this subdomain focus on the reliability of methods involving GA research. In favor of restrictions, authors argued that the methodology used in current GA research is questionable because, for example, GA experiments can produce misleading results due to the multiple diseases that GA used in research tend to have, or due to the stress GA face in laboratory life. Divergent views were expressed as to whether the phylogenetic continuity, or apparent genetic similarity, between GA and humans implies that GA are good animal models for studying human diseases.

#### Welfare

Many reasons for and against restricting invasive GA research focused on GA welfare in research facilities (22 mentions in favor and 10 against). Within this domain, there was disagreement in three main areas: (1) care and housing requirements, (2) conditions in research facilities, and (3) harms caused by GA research. In terms of care and housing, authors favoring restrictions argued that the exacting requirements of GA care and housing are not, or cannot be, fulfilled while authors arguing against restrictions put forward the opposite claim.

With respect to the conditions of GA in research facilities, authors disagreed as to whether the conditions of captive GA are adequate. Authors favoring restrictions contended that captivity could cause GA psychological harms or alter their normal development. In contrast, authors arguing against restrictions contended that GA are better off (e.g., in terms of life-expectancy or wellbeing) in research facilities than they would be otherwise (e.g., in the wild, or if they were abandoned by their owners).

Regarding harms to GA resulting from research activities, authors in favor of restrictions claimed that GA research sometimes significantly harms GA both physically and psychologically. Authors also pointed to the harms GA suffer by virtue of being long-lived: for instance, that they can be kept in laboratories for decades while being used for multiple research protocols. Against restrictions, authors argued that GA research can be carried out without significantly harming GA, and that GA research is necessary for improving GA’s own welfare.

#### Public and expert attitudes

Reasons appealing to public and expert attitudes were all used in favor of restricting invasive research with GA (24 mentions).^5^ The most frequently cited reason was that many (especially scientifically advanced) countries have already restricted GA biomedical research. Authors also indicated as reasons the decrease in public, scientific, or institutional support, or the growing opposition to the use of GA in research.

#### Conservation and retirement

Reasons related to conservation and retirement constitute the only domain in which there is a clear predominance of reasons against over those in favor of restricting GA research (6 mentions in favor and 14 against). Reasons in this domain were more common in scientific (especially health sciences) journals. Authors arguing for restrictions—especially those writing until the mid-1990s, when chimpanzees bred in captivity were still considered scarce—claimed that supplying GA for research has led to a decline of wild populations and the threat of extinction. More recently, with the abundance of chimpanzees bred in captivity, some authors pointed to the continued endangered status of GA to argue in favor of restrictions. Conversely, some authors appealed to the availability of GA in research facilities as an argument in favor of continuing GA research. Furthermore, some argued that GA research could benefit GA as a species on grounds that it could assist conservation efforts (e.g., by improving the survival, welfare, and protection of the species).

Another point of contention regarding research use of GA relates to their retirement, especially given both that GA are long-lived and that euthanasia is generally considered inappropriate in or following GA research. Against restrictions, authors argued that GA could be cared for post-experiments in other research facilities or in alternative indoor/outdoor facilities (e.g., sanctuaries). In favor of restrictions, one author contended that optimal GA retirement would involve returning them to the wild, which is not feasible, especially for GA brought up in captivity (e.g., because they have been deprived from the opportunity to learn the skills needed for surviving in the forest).

#### Respect and rights

Reasons in this domain appeal to the notions of consent, personhood, and rights and were used solely to argue in favor of restricting GA research (15 mentions). There was some overlap between the reasons in this domain and the reasons included in the Moral Standing domain; however, reasons in this domain were more explicit and suggested that it is wrong to treat GA in certain ways, irrespective of the harms that such treatment may cause. Most of the reasons in this domain appeared in SS&H journals.

Authors appealed to the widely accepted idea that respect for research subjects requires consent when appropriate and argued that, like children, GA are capable of assenting or dissenting. Others claimed that GA should be protected because they can be considered subjects with diminished or no capacity for informed consent. Some authors appealed to the notion of personhood, arguing that GA possess enough cognitive capacities to be considered persons (or near-persons, or person-like). As for moral rights, a few authors argued that since GA possess the capacities we take as grounding moral rights (e.g., the right to freedom or welfare), we should regard GA as having these rights.

#### Financial costs

Reasons in this domain appeal to the financial costs of GA research (10 mentions in favor and 3 against). Almost all these reasons were published in scientific journals. In favor of restrictions, many argued that the financial costs required for GA care and housing are too high for GA research to be cost-effective. Similarly, some authors drew attention to the financial burden associated with conducting GA research, including providing them with long-term care after research (which can last for several decades). Against restrictions, authors argued that the costs of long-term care are manageable, or that GA research could be affordable (e.g., by using a small number of animals) and cost-effective in the long-term (e.g., by reducing the costs of human healthcare).

#### Law and legal status

Considerations involving the law and legal status of GA were mostly used to argue in favor of restrictions on invasive GA research (9 mentions in favor and 2 against). The distinction between legal reasons and moral reasons is important because they are not coextensive: one could argue, for example, that GA should be granted the legal right to life without arguing that they also have a moral right to life. Law and Legal Status is the domain in which the largest proportion of mentions came from SS&H journals. Authors appealed to laws and policies already in place to argue both for and against restrictions. In favor of restrictions, some authors argued that laws and policies already restrict the use of GA for research, while another argued against restrictions by stating that laws and policies protecting GA vary in terms of strictness depending on setting (e.g., research, zoos, or private homes). Authors also disagreed about whether to grant GA legal personhood, which would entail conceding them basic legal rights on a par with those of humans. Some argued that, given their cognitive capacities, GA should be granted legal personhood, whereas one author warned that granting GA legal personhood would place society on a slippery slope leading to granting legal personhood to other animals.

With respect to granting GA legal rights, a single article made the following four claims: that GA should be granted legal rights to liberty, to personal security, to life, and not to be subjected to experiments that are not in their best interests. No corresponding reasons against restrictions were offered.

#### Longer-term consequences

This domain comprises reasons that appealed to the consequences of GA research in the longer-term (e.g., for other animals, or for public attitudes about research on GA or other animals) (5 mentions in favor and 2 against). In favor of restrictions, some argued that restricting GA research is instrumental to restrict research on other animals, or that it is an important first step away from speciesism against GA. One point of contention was whether restricting invasive GA research would have a negative impact on non-invasive (e.g., behavioral and observational) GA research, due to the decreasing number of GA kept in colonies.

## Discussion

This is the first systematic review addressing the ethics of regulating research with some animal species. We provided a comprehensive review of academic articles on the issue of restricting biomedical research with GA. We identified a total of 110 reason-types, of which 74 were in favor of and 36 were against restricting GA research. Previous articles on this topic and in our review offered a maximum of 15 reason types [cf. 26]. This review thus offers the most comprehensive overview of the current debate, as well as a unique analysis of the arguments put forward in the academic literature. It is also the first systematic review of reasons that identifies articles posing potential conflicts of interest.

While this review does not attempt to settle the question of whether or not invasive research on GA should be restricted, it reveals several important insights, both for advancing this debate and for advancing the debate on the use of animals more generally in invasive research. As a result, it can help policymakers make informed decisions, with minimal bias, concerning possible restrictions to GA research and research with other animal species.

### An academic trend that parallels a regulatory trend toward restrictions

Our findings suggest a trend in the ethical debate in favor of restricting GA research that parallels both a more general social and institutional trend and worldwide policy changes in the same direction. The use of GA for research began in the first half of the twentieth century, but it drastically increased in the 1980s, prompted by the AIDS epidemic [72]. Seemingly in response to this spike in GA research, the academic and public debate on the ethics of this research increased both in the 1980s and in the 1990s, when the population of chimpanzees in laboratories peaked. This coincides with the U.K.’s 1997 ban on GA research, which was followed by similar restrictions in many countries in Europe and Oceania [46]. The academic debate increased again in the years surrounding 2011, when the Institute of Medicine (IOM) issued a report recommending more demanding standards on the use of chimpanzees in research. That previous year, E.U. Directive 2010/63 mandated a ban on the use of GA, which went into effect in 2013. In 2015, in response to the IOM’s report, the NIH decided to end the invasive use of chimpanzees in research (see Fig. 2).

Overall, more than three-quarters of the articles in our review argued for increasing restrictions on, or banning, GA research. A similar trend appeared with respect to the reason-types offered in the literature: the number of reason-types in favor of restrictions was twice the number of reasons against restrictions. The former were also mentioned much more frequently than the latter (239 vs 77 mentions). The NIH’s 2015 decision to end invasive chimpanzee research virtually ended such research worldwide and has been followed by a decline in the academic debate. Only two articles in our review were published after 2016.

### Some domains were rather one-sided

Moral Standing had the highest number of mentions, but reasons in this domain were rather one-sided. Fifteen reasons comprising 103 mentions in favor of restricting GA research were met with just one mention of one reason against. Most reasons within this domain appealed to the cognitive, consciousness-related, or behavioral capacities of GA to argue in favor of restrictions. Though rarely made explicit, the rationale behind these reasons was seemingly the idea that these capacities ground higher moral standing, and that this standing is crucial in determining whether we should restrict or ban GA research. (“*Higher* moral standing” here might mean either higher than has traditionally been assumed or higher than the moral standing of most or all other nonhuman animals.) Although the implicitness of this rationale may stem from a wider consensus that GA have higher moral standing by virtue of their complex or sophisticated capacities, the rationale itself does raise some questions that were not addressed in the debate. In particular, is moral standing the right kind of ground for restricting or banning GA research? Moreover, do cognitive, consciousness-related, or behavioral capacities ground moral standing in the first place? The scholarly debate might therefore advance if authors engage more explicitly with the reasoning behind three main ideas: (1) that GA have higher moral standing (in either of the two senses identified above); (2) that GA’s moral standing warrants them protections that are not warranted for other animal groups (much as human beings’ moral standing is assumed to warrant special protections for human subjects); and (3) that GA’s cognitive, consciousness-related, and/or behavioral capacities are the basis for their moral standing. Authors might also wish to explore whether moral standing-related reasons could extend to other animal species, thereby addressing the issue of whether GA have higher moral standing than other research animals.

Another domain characterized by one-sidedness in the reasons offered is Respect and Rights, in which all reasons favored restrictions (6 reasons, 15 mentions). Appeals to cognitive, consciousness-related, or behavioral capacities as grounds for affording GA special protections were also common in this domain. But, unlike the reasons presented in Moral Standing, these reasons were more explicit and elaborate in that they appealed to thicker ethical concepts as personhood, respect, and rights. The greater explicitness and elaboration characterizing these arguments might be explained by the fact that these reasons were published primarily in peer-reviewed articles in SS&H journals. The domain Respect and Rights offers fertile ground for advancing the debate on the ethics of GA research insofar as more developed arguments for restrictions encourage well-developed counterarguments and, over time, clearer illumination of the relevant issues. Moreover, claims about personhood, respect, and rights might have implications for discussions in the domain of Law and Legal Status, which contains reasons regarding granting legal personhood to chimpanzees. Overall, more in-depth discussions regarding these thick ethical concepts, along with fuller exploration of opposing viewpoints, hold promise for advancing the debate.

### Some domains revealed significant engagement between opposing views

Two domains featured considerable engagement between positions in favor of and against restricting GA research: Science and Welfare. These domains, together with Moral Standing, contained two-thirds of all of the reason mentions in our review, but unlike the debate in Moral Standing, the debate in the domains Science and Welfare seems to have had a chance to mature. The convergence of reasons in these domains may reflect an emerging trend in the animal ethics debate, with authors arguing that traditional regulatory frameworks (and the “Three Rs” that serve as their foundation) are insufficient to ensure the value of animal research for improving health and wellbeing, especially in relation to research costs, and is insufficiently protective of the welfare of laboratory animals [73, 74]. After all, many of the reasons in these domains concerned the scientific or medical value of GA research and the harms it causes to GA.

Engagement between opposing reasons, albeit with fewer mentions, was also apparent in three other domains: Conservation and Retirement, Financial Costs, and Law and Legal Status. Interestingly, all of these domains raised concerns particularly relevant to GA. For example, in Conservation and Retirement, reasons concerned the fact that GA are scarce, endangered species; in Financial Costs reasons highlighted the high costs associated with GA housing and long-term care; and in Law and Legal Status reasons raised concerns about the legal status of GA—concerns that do not generally arise for other animals. Insofar as the debate surrounding GA research has declined due to worldwide restrictions, we may not expect much more development on these domains as they apply to GA. But they represent interesting points of discussion that may be extended to debates involving the use of other animal species, on a case by case basis.

### Diverse, independent views were lacking

Overall, our review revealed low diversity and independence among the views presented in the literature. For example, most articles (72%) defending a strong position (i.e., favoring a total ban or arguing against restrictions) were associated with institutions that we identified as taking a position on GA research or animal research more generally (which we identified as posing potential conflicts of interests; see Additional file 1: Table 2). Moreover, all U.K.-based authors argued in favor of a total ban, which is unsurprising given that the U.K. was a pioneer in phasing out GA research. Similarly, all authors arguing against restrictions were based in two of the handful of countries where GA research was conducted at the time the articles were written: the U.S. (12) and The Netherlands (1).

This phenomenon, together with the aforementioned one-sidedness in some domains, is concerning. The debate on animal research has long been perceived as locked into polarized and sometimes oversimplified positions, with neither side listening to the other. The scholarly debate on restricting GA research might thus advance if the interaction between academics and the public is reimagined as a collaborative effort, in which the public shapes the debate by organizing and voicing widely-shared concerns and ideals, both in favor of and against restrictions, and academics step forward to clarify, evaluate, restructure, and strengthen arguments that might otherwise be weak or incomplete. In brief, we might see the role of activists as raising awareness of important concerns and issues and of academics (either as observers or participants in advocacy movements) as adding rigor and balance to the ongoing debate [75]. In this process and generally, authors should be cognizant of, and forthcoming about, potential conflicts of interests when arguing for or against restrictions on GA research.

### Scholarly discussion was not the norm

Overall, almost half of the articles in our final list were opinion pieces or conference proceedings—publications that are less likely to have been peer-reviewed. This contrasts with previous systematic reviews of reasons in which the proportion of non-peer-reviewed articles was much smaller [76]. Even though scholarly bioethics articles are sometimes not peer-reviewed for editorial reasons (e.g., in some scientific journals), our findings suggest that scholarly discussion has not been the norm.

A further contrast with previous reviews, in which authors were primarily affiliated with academic institutions [77], is that only one-third of corresponding authors in the present review had such an affiliation, while over half were affiliated with a private nonacademic institution or foundation. These facts underscore both a tendency away from scholarly discussion and the impact that private nonacademic institutions or foundations, including advocacy groups, can have both on shaping scholarly debates and on generating policy changes.

The tendency away from scholarly discussion may be further supported by the common use of persuasion tactics that, while effective for advancing political commitments, may reflect errors in reasoning. For example, authors favoring restrictions frequently appealed to popular sentiment, decisions by particular countries or industries, or expert attitudes: there were 25 such mentions, and no mentions against restricting GA research. Yet, while these appeals may reflect a general social and institutional trend towards stronger protections for GA—which may be a legitimate concern for those affected (e.g., taxpayers)—they may be interpreted as committing what in logic is called the fallacy of appeal to authority. Many authors seem also to have engaged in what can be considered a one-sidedness fallacy—that is, presenting evidence for a certain viewpoint with little or no effort to engage with opposing viewpoints. This is evidenced by the fact that only 2 (3%) of the articles in our entire review cited other articles in our review to engage critically with an opposing viewpoint.

While it is not the place of a systematic review of reasons to measure the quality of the reasons presented in the literature, our findings nonetheless highlight the need for a fairer and more balanced scholarly discussion.

### Future directions

Given the clear academic and policy trends toward increasing restrictions on, or banning, GA research, it seems extremely likely to us that the trend will continue rather than reverse direction. From the standpoint of those who believe such a trend is justified rather than misguided, the growing recognition that animals have substantial moral standing may appear to reflect a form of moral progress. However, from the standpoint of those who do not believe this trend is justified, there are many avenues for arguments that may alter the direction of this trend. Either way, one natural avenue for future research is to explore, in light of the reasons offered in this review, whether animals other than GA deserve greater research protections than they currently enjoy.

How plausible would it be to extend the reasons put forward in the context of the GA debate in judging whether invasive research on other animal species should be restricted? In some respects, GA represent a singular case for creating restrictions on research use. Some reasons that seem to apply well in the case of GA are for example those within the domains Public and Expert Attitudes, Conservation and Retirement, and Financial Costs, but may not apply to animal species that are, say, less popular among the public, more widespread and abundant, or less expensive to house and care for. Moreover, certain reasons that were successful in advancing the case for GA restrictions such as appeals to cognitive capacities, similarity to humans, or welfare requirements may only work in a limited set of cases, such as other primates, but excluding, say, rodents.

Further avenues for advancing the debate come from concerns not found in this review, such as appeals to moral virtues and vices, animal flourishing, and special relationships, which may be worth exploring in relation to the ethics of research with GA or other animal species or groups. One notable example is the National Academies of Sciences, Engineering, and Medicine report on canines, which concludes that in certain qualified cases, it is justified to give dogs preferential treatment by virtue of our close relationship and societal preference towards them [6]. Finally, it is worth noting that reasons identified in this review were predominantly deontological—that is, focused on rights, duties, or obligations regarding our treatment to GA—rather than consequentialist. With the important exception of the domain of Science (in which the value of research for society was a key consideration), there was scant systematic discussion of the consequences of restricting or not restricting GA research. Thus, the domain Longer-term Consequences may provide an interesting path for future work, addressing, for example, whether restricting, or not restricting, the use of certain animal species for research may affect other animals, humans, or the environment.

### Limitations

Some limitations of this review must be acknowledged. As we excluded articles that were not published in English, it is possible that this review overlooks arguments in the non-English literature on regulating GA research. However, English has become the dominant language in bioethics publications, which suggests that our review is representative of the concerns raised in the literature.

The restriction to journal articles—common in systematic reviews in bioethics—might also lead to the exclusion of certain reasons present in other kinds of publications (e.g., textbooks). However, it should be noted that at final stages of data analysis (i.e., based on ten articles added after perusal of reference lists) our results reached considerable saturation and no new (sub)domains had to be created.

Another potential limitation involves the different types of articles included in this review. Insofar as we included opinion pieces (e.g., commentaries and letters), it is possible that the quality of the reasons offered is lower than those published in a peer-reviewed journal. However, we tried to be as systematic and comprehensive as possible in a field in which explicit ethical arguments were often put forward in different types of articles, especially in scientific journals. Moreover, given their impact on policy, excluding expert opinion pieces would have skewed our review.

A final limitation concerns our categorization: although we grouped reasons based on their content, and with an eye towards being informative to the reader, we recognize that some reasons could have been grouped differently. For example, although no consensus exists on how the notions of *harms* and *wrongs* should be distinguished, we separated reasons involving the notions of respect, personhood, and rights—notions often associated with *wronging*—from reasons involving the cognitive, consciousness-related, or behavioral capacities of GA, which are often associated with the potential to suffer considerable harm.

## Conclusion

Over the last three decades, the use of GA in invasive biomedical research has generated widespread concern, both academically and among the public, as well as worldwide policy changes in favor of restrictions. This review shows that the most debated areas in this topic discuss the scientific and medical value of GA research as well as the welfare of GA in research. This is consistent with current trends in the animal ethics literature. Still, the most frequently mentioned reasons made direct or indirect appeal to the moral standing of GA. These reasons were used almost exclusively to argue in favor of restrictions and offer several avenues for advancing the debate.

Authors rarely cited other articles defending an opposing viewpoint and many domains revealed one-sided concerns. Moreover, our findings suggest that non-academic institutions or foundations, including advocacy groups, have figured prominently in this debate. This is suggested both by the high number of nonacademic articles that met the criteria to be included in our final list, and by the high number of potential conflicts of interests among publications included in this review, especially those favoring a strong position either in favor of or against restrictions. Our findings also call for more transparency and diversity in the debate on GA research, thereby promoting opportunities to foster collaborative efforts between academics and the public—efforts that could move the debate forward. Insofar as the trend towards increasing restrictions on GA research is likely to continue and extend to other animal species or groups, this review offers a clear and detailed map of the debate as well as promising avenues for advancing it.

## Supplementary Information

**Additional file 1: Tables 1, 2**. Final search strategies used (Table 1), and journal or institution associated with potential conflicts of interest (Table 2)

## Data Availability

The datasets generated and analyzed for this article are available from the corresponding author on reasonable request.

## Abbreviations

GA: Great apes
PRISMA: Preferred reporting items for systematic reviews and meta-analyses
ASJC: All Science journal classification
SS&H: Social sciences & humanities
HIV: Human immunodeficiency virus
AIDS: Acquired immunodeficiency syndrome
IOM: Institute of medicine

## Footnotes

1 To be more precise, great apes, or hominids, comprise seven species of non-human great apes: Pan troglodytes (chimpanzees); Pan paniscus (bonobos); Gorilla gorilla and Gorilla beringei (gorillas); and Pongo abelii, Pongo pygmaeus, and Pongo tapanuliensis (orangutans). Humans are also considered great apes, but since our review focuses on biomedical research with non-human animals, we use ‘great apes’ to denote the non-human species just mentioned.

2 As we explain later, by ‘invasive biomedical research’ we mean research that is potentially harmful and not primarily aimed to benefit the individual or the species to which it belongs.

3 Roughly, health sciences journals cover research on human health, disease and health professions; life sciences journals cover biological and animal research; and social sciences & humanities journals cover different branches of those disciplines, including bioethics. To simplify the exposition, we sometimes refer to health sciences and life sciences journals together as ‘scientific journals’. No publication included in our review corresponded to a physical sciences journal.

4 Within articles identified as posing a potential conflict of interests, both articles favoring restrictions and articles against restrictions were associated with institutions taking the same position towards animal research.

5 Note that although we did not include appeals to others’ claims as reasons in this review, the reasons advanced in this domain were not simply appeals to others’ views; they were actively used by authors to support their own arguments. We elaborate on this point in the Discussion.
