# Supplementary material for: Should biomedical research with great apes be restricted? A systematic review of reasons
Source: BMC Med Ethics. 2021 Feb 16;22:15. doi: 10.1186/s12910-021-00580-z (PMC7888082; doi:10.1186/s12910-021-00580-z)
Supplement: Supplementary file 1 — Additional file 1: Tables 1, 2. Final search strategies used (Table 1), and journal or institution associated with potential conflicts of interest (Table 2) [file 12910_2021_580_MOESM1_ESM.docx]

**Additional files Table 1**. Final search strategies used.

| **Database:** PubMed  **Vendor:** National Library of Medicine  **Date searched:** July 14, 2020  **Limits used:** Language: English |
| --- |
| ("Gorilla gorilla"[Mesh] OR "Pongo"[Mesh] OR "Pan paniscus"[Mesh] OR "Pan troglodytes"[Mesh] OR gorilla[tw] OR gorillas[tw] OR chimpanzee[tiab] OR chimpanzees[tiab] OR chimps[tiab] OR orangutan[tiab] OR orangutans[tiab] OR “great ape”[tiab] OR “great apes”[tiab] OR bonobos[tiab] OR bonobo[tiab] OR troglodytes[tiab] OR pongidae[tw] OR pongo[tw] OR "pan troglodytes"[tw] OR "orang utans"[tiab]) AND ("Biomedical Research"[Mesh] OR "Animal Experimentation"[Mesh] OR “biomedical research”[tiab] OR “investigational medicine”[tiab] OR “experimental medicine”[tiab] OR “experimental research”[tiab] OR “investigative medicine”[tiab] OR “medical research”[tiab] OR “animal experimentation”[tiab] OR “animal research”[tiab] OR “animal experiment”[tiab] OR “animal experiments”[tiab] OR “invasive research”[tiab] OR “harmful research”[tiab] OR experimentation[tiab] OR invasive[tiab] OR “Animals, Laboratory”[mesh] OR “Animal Welfare”[mesh]) AND (ethics[tiab] OR ethical[tiab] OR Ethics[mesh] OR Ethics, Research[mesh] OR Bioethics[mesh] OR ethics[subheading] OR bioethics[tiab] OR regulatory[tiab] OR morals[tiab] OR regulation[tiab] OR regulations[tiab] OR regulated[tiab] OR legal[tiab] OR legality[tiab] OR legalities[tiab] OR legislation[tiab] OR legislate[tiab] OR legislates[tiab] OR legislated[tiab] OR policy[tiab] OR policies[tiab] OR law[tiab] OR laws[tiab] OR ban[tiab] OR bans[tiab] OR banned[tiab] OR limitation[tiab] OR limitations[tiab] OR restrict[tiab] OR restriction[tiab] OR restrictions[tiab] OR moratoria[tiab] OR moratorium[tiab] OR moratoriums[tiab] OR embargo[tiab] OR embargoes[tiab] OR embargoed[tiab] OR prohibit[tiab] OR prohibits[tiab] OR prohibited[tiab] OR prohibition[tiab] OR prohibitions[tiab] OR suspend[tiab] OR suspension[tiab] OR suspensions[tiab] OR suspends[tiab] OR suspended[tiab] OR stoppage[tiab] OR halt[tiab] OR halts[tiab] OR halted[tiab] OR halting[tiab] OR freeze[tiab] OR frozen[tiab] OR protection[tiab] OR protections[tiab] OR protect[tiab] OR protected[tiab] OR protects[tiab] OR forbid[tiab] OR forbids[tiab] OR forbidden[tiab] OR Public Policy[mesh]) AND English[lang] |
|  |
| **Database:** Web of Science: Science Citation Index; Social Sciences Citation Index; Book Citation Index SCI; Book Citation Index: SSH  **Vendor:** Clarivate Analytics  **Date searched:** July 14, 2020  **Limits:** English language only; Use Topic to limit search to this field; Document type: limit to these fields only: Article, Book, Book Chapter, Discussion, Early Access, Editorial Material, Letter, reprint, Review  **Notes:** Use Advanced search. Run main search first. Then go back and apply the language filter to select English only, and the Document Types specified. |
| TS=(("Gorilla gorilla" OR Pongo OR "Pan paniscus" OR "Pan troglodytes" OR gorilla OR gorillas OR chimpanzee OR chimpanzees OR chimps OR orangutan OR orangutans OR “great ape” OR “great apes” OR bonobos OR bonobo OR troglodytes OR pongidae OR "orang utans") AND ("Biomedical Research" OR "Animal Experimentation" OR “investigational medicine” OR “experimental medicine” OR “experimental research” OR “investigative medicine” OR “medical research” OR “animal research” OR “animal experiment” OR “animal experiments” OR “invasive research” OR “harmful research” OR experimentation OR invasive OR “laboratory animals” OR “Animal Welfare”) AND (ethics OR ethical OR Bioethics OR regulatory OR morals OR regulation OR regulations OR regulated OR legal OR legality OR legalities OR legislation OR legislate OR legislates OR legislated OR policy OR policies OR law OR laws OR ban OR bans OR banned OR limitation OR limitations OR restrict OR restriction OR restrictions OR moratoria OR moratorium OR moratoriums OR embargo OR embargoes OR embargoed OR prohibit OR prohibits OR prohibited OR prohibition OR prohibitions OR suspend OR suspension OR suspensions OR suspends OR suspended OR stoppage OR halt OR halts OR halted OR halting OR freeze OR frozen OR protection OR protections OR protect OR protected OR protects OR forbid OR forbids OR forbidden OR “Public Policy”)) |
|  |
| **Database:** Web of Science: BIOSIS Citation Index  **Vendor:** Clarivate Analytics  **Date searched:** July 14, 2020  **Limits:** English language only; Use Topic to limit search to this field; Document type: limit to these fields only: Article, Book, Book Chapter, Letter, Reprint, Technical Report.  **Notes:** Use Advanced search. Run main search first. Then go back and apply the language filter to select English only, and the Document Types specified. |
| TS=(("Gorilla gorilla" OR Pongo OR "Pan paniscus" OR "Pan troglodytes" OR gorilla OR gorillas OR chimpanzee OR chimpanzees OR chimps OR orangutan OR orangutans OR “great ape” OR “great apes” OR bonobos OR bonobo OR troglodytes OR pongidae OR "orang utans") AND ("Biomedical Research" OR "Animal Experimentation" OR “investigational medicine” OR “experimental medicine” OR “experimental research” OR “investigative medicine” OR “medical research” OR “animal research” OR “animal experiment” OR “animal experiments” OR “invasive research” OR “harmful research” OR experimentation OR invasive OR “laboratory animals” OR “Animal Welfare”) AND (ethics OR ethical OR Bioethics OR regulatory OR morals OR regulation OR regulations OR regulated OR legal OR legality OR legalities OR legislation OR legislate OR legislates OR legislated OR policy OR policies OR law OR laws OR ban OR bans OR banned OR limitation OR limitations OR restrict OR restriction OR restrictions OR moratoria OR moratorium OR moratoriums OR embargo OR embargoes OR embargoed OR prohibit OR prohibits OR prohibited OR prohibition OR prohibitions OR suspend OR suspension OR suspensions OR suspends OR suspended OR stoppage OR halt OR halts OR halted OR halting OR freeze OR frozen OR protection OR protections OR protect OR protected OR protects OR forbid OR forbids OR forbidden OR “Public Policy”)) |
|  |
| **Database:** Web of Science: Zoological Record  **Vendor:** Clarivate Analytics  **Date searched:** July 14, 2020  **Limits:** English language only; Use Topic to limit search to this field; Document type: limit to these fields only: Article, Book, Book Chapter.  **Notes:** Use Advanced search. Run main search first. Then go back and apply the language filter to select English only, and the Document Types specified. |
| TS=(("Gorilla gorilla" OR Pongo OR "Pan paniscus" OR "Pan troglodytes" OR gorilla OR gorillas OR chimpanzee OR chimpanzees OR chimps OR orangutan OR orangutans OR “great ape” OR “great apes” OR bonobos OR bonobo OR troglodytes OR pongidae OR "orang utans") AND ("Biomedical Research" OR "Animal Experimentation" OR “investigational medicine” OR “experimental medicine” OR “experimental research” OR “investigative medicine” OR “medical research” OR “animal research” OR “animal experiment” OR “animal experiments” OR “invasive research” OR “harmful research” OR experimentation OR invasive OR “laboratory animals” OR “Animal Welfare”) AND (ethics OR ethical OR Bioethics OR regulatory OR morals OR regulation OR regulations OR regulated OR legal OR legality OR legalities OR legislation OR legislate OR legislates OR legislated OR policy OR policies OR law OR laws OR ban OR bans OR banned OR limitation OR limitations OR restrict OR restriction OR restrictions OR moratoria OR moratorium OR moratoriums OR embargo OR embargoes OR embargoed OR prohibit OR prohibits OR prohibited OR prohibition OR prohibitions OR suspend OR suspension OR suspensions OR suspends OR suspended OR stoppage OR halt OR halts OR halted OR halting OR freeze OR frozen OR protection OR protections OR protect OR protected OR protects OR forbid OR forbids OR forbidden OR “Public Policy”)) |
|  |
| **Database:** Global Health  **Vendor:** CAB Direct  **Date searched:** July 14, 2020  **Limits:** English language only; Use title and abstract to limit search to these fields; Document type: limit to these fields only: Journal Article, Journal Issue, Book.  **Notes:** Use Advanced search. Run main search first. Then go back and apply the language filter to select English only, and the Document Types specified. |
| #1 Title: ("Gorilla gorilla" OR Pongo OR "Pan paniscus" OR "Pan troglodytes" OR gorilla OR gorillas OR chimpanzee OR chimpanzees OR chimps OR orangutan OR orangutans OR “great ape” OR “great apes” OR bonobos OR bonobo OR troglodytes OR pongidae OR "orang utans")  #2 Abstract: ("Gorilla gorilla" OR Pongo OR "Pan paniscus" OR "Pan troglodytes" OR gorilla OR gorillas OR chimpanzee OR chimpanzees OR chimps OR orangutan OR orangutans OR “great ape” OR “great apes” OR bonobos OR bonobo OR troglodytes OR pongidae OR "orang utans")  #3 Subject Term: "Gorilla" OR "Gorilla gorilla" OR "gorillas" OR "Pongidae" OR "Pan" OR "Pongo" OR "Pan paniscus" OR "chimpanzees"  #4 #1 OR #2 OR #3  #5 Title: ("Biomedical Research" OR "Animal Experimentation" OR “investigational medicine” OR “experimental medicine” OR “experimental research” OR “investigative medicine” OR “medical research” OR “animal research” OR “animal experiment” OR “animal experiments” OR “invasive research” OR “harmful research” OR experimentation OR invasive OR “laboratory animals” OR “Animal Welfare”)  #6 Abstract: ("Biomedical Research" OR "Animal Experimentation" OR “investigational medicine” OR “experimental medicine” OR “experimental research” OR “investigative medicine” OR “medical research” OR “animal research” OR “animal experiment” OR “animal experiments” OR “invasive research” OR “harmful research” OR experimentation OR invasive OR “laboratory animals” OR “Animal Welfare”)  #7 Subject Term: "animal experiments" OR "animal testing alternatives" OR "laboratory animals" OR "animal research" OR "medical research"  #8 #5 OR #6 OR #7  #9 Title: (ethics OR ethical OR Bioethics OR regulatory OR morals OR regulation OR regulations OR regulated OR legal OR legality OR legalities OR legislation OR legislate OR legislates OR legislated OR policy OR policies OR law OR laws OR ban OR bans OR banned OR limitation OR limitations OR restrict OR restriction OR restrictions OR moratoria OR moratorium OR moratoriums OR embargo OR embargoes OR embargoed OR prohibit OR prohibits OR prohibited OR prohibition OR prohibitions OR suspend OR suspension OR suspensions OR suspends OR suspended OR stoppage OR halt OR halts OR halted OR halting OR freeze OR frozen OR protection OR protections OR protect OR protected OR protects OR forbid OR forbids OR forbidden OR “Public Policy”)  #10 Abstract: (ethics OR ethical OR Bioethics OR regulatory OR morals OR regulation OR regulations OR regulated OR legal OR legality OR legalities OR legislation OR legislate OR legislates OR legislated OR policy OR policies OR law OR laws OR ban OR bans OR banned OR limitation OR limitations OR restrict OR restriction OR restrictions OR moratoria OR moratorium OR moratoriums OR embargo OR embargoes OR embargoed OR prohibit OR prohibits OR prohibited OR prohibition OR prohibitions OR suspend OR suspension OR suspensions OR suspends OR suspended OR stoppage OR halt OR halts OR halted OR halting OR freeze OR frozen OR protection OR protections OR protect OR protected OR protects OR forbid OR forbids OR forbidden OR “Public Policy”)  #11 Subject Term: "ethics" OR "bioethics" OR "regulations" OR "legislation"  #12 #9 OR #10 OR #11  #13 #4 AND #8 AND #12 |
| **Database:** EthxWeb <https://repository.library.georgetown.edu/handle/10822/761853>  **Vendor:** Georgetown University Library  **Date searched:** July 14, 2020  **Limits:** None  **Notes:** Use the “search within this collection” option to conduct each search |
| Search #1: (ethics OR ethical OR Bioethics OR regulatory OR morals OR regulation OR regulations OR regulated OR legal OR legality OR legalities OR legislation OR legislate OR legislates OR legislated OR policy OR policies OR law OR laws OR ban OR bans OR banned OR limitation OR limitations OR restrict OR restriction OR restrictions OR moratoria OR moratorium OR moratoriums OR embargo OR embargoes OR embargoed OR prohibit OR prohibits OR prohibited OR prohibition OR prohibitions OR suspend OR suspension OR suspensions OR suspends OR suspended OR stoppage OR halt OR halts OR halted OR halting OR freeze OR frozen OR protection OR protections OR protect OR protected OR protects OR forbid OR forbids OR forbidden OR “Public Policy”) AND ("Biomedical Research" OR "Animal Experimentation" OR “investigational medicine” OR “experimental medicine” OR “experimental research” OR “investigative medicine” OR “medical research” OR “animal research” OR “animal experiment” OR “animal experiments” OR “invasive research” OR “harmful research” OR experimentation OR invasive OR “laboratory animals” OR “Animal Welfare”) AND ("Gorilla gorilla" OR Pongo OR "Pan paniscus" OR "Pan troglodytes" OR gorilla OR gorillas OR chimpanzee OR chimpanzees OR chimps OR orangutan OR orangutans OR “great ape” OR “great apes” OR bonobos OR bonobo OR troglodytes OR pongidae OR "orang utans")  Search #2: “great apes”  Search #3: "great apes" OR gorilla* OR chimpanzee* OR organutan*  Search #4: “great ape” NOT ("great apes" OR gorilla* OR chimpanzee* OR organutan |
|  |
| **Database:** PhilPapers <https://philpapers.org/>  **Vendor:** Centre for Digital Philosophy **Date searched:** July 14, 2020  **Limits:** None. **Notes:** Use Advanced Search and then either the Fuzzy Filter Advanced or Fuzzy Filter Basic, and check mark the professional authors and published only options. For Fuzzy Filter Advanced, check mark the “Append words from mandatory sets (above) to this list” option. |
| Search #1: Advanced Search > Fuzzy Filter Advanced > Must appear: "great apes" \| “great ape” \| gorilla* \| chimpanzee* \| orangutan* & professional authors & published only  Search #2: Advanced Search > Fuzzy Filter Basic: ("great apes" \| "great ape" \| gorilla \| chimpanzee \| orangutan) & ("animal experimentation" \| “investigational medicine” \| “experimental medicine” \| “experimental research” \| “investigative medicine” \| “medical research” \| “animal research” \| “animal experiment” \| “animal experiments” \| “invasive research” \| “harmful research”) & check mark “Append words from mandatory keyword sets (above) to this list” & professional authors & published only  Search #3: Advanced Search > Fuzzy Filter Basic: ("great apes" \| gorilla \| chimpanzee \| orangutan) & (ethic \| regulation \| legal \| law \| moratorium \| suspend) & check mark “Append words from mandatory keyword sets (above) to this list” & professional authors & published only  Search #4: Advanced Search > Fuzzy Filter Basic: (ethics \| ethical \| Bioethics \| regulatory \| morals \| regulation \| regulations \| regulated \| legal \| legality \| legalities \| legislation \| legislate \| legislates \| legislated \| policy \| policies \| law \| laws \| ban \| bans \| banned \| limitation \| limitations \| restrict \| restriction \| restrictions \| moratoria \| moratorium \| moratoriums \| embargo \| embargoes \| embargoed \| prohibit \| prohibits \| prohibited \| prohibition \| prohibitions \| suspend \| suspension \| suspensions \| suspends \| suspended \| stoppage \| halt \| halts \| halted \| halting \| freeze \| frozen \| protection \| protections \| protect \| protected \| protects \| forbid \| forbids \| forbidden \| “Public Policy”) & ("great apes" \| gorilla \| chimpanzee \| orangutan) & check mark “Append words from mandatory keyword sets (above) to this list” & professional authors & published only |

**Additional files Table 2**. Journal or institution associated with potential conflicts of interest.

| **Entity** | **Position** | **Name** | **Statement** | **Link** |
| --- | --- | --- | --- | --- |
| **Journal** | Pro | Alternatives to Laboratory Animals (ATLA) | From sponsor (FRAME): FRAME’s ultimate aim is the elimination of the need to use laboratory animals in any kind of medical or scientific procedures. | <https://frame.org.uk/researching-alternatives-to-animal-testing/vision-values/> |
|  | Con | National Society for Medical Research | The National Society for Medical Research was established in 1946 with the purpose of ensuring the freedom of investigators and teachers to use laboratory animals, whenever such use is justified. | <https://oculus.nlm.nih.gov/cgi/f/findaid/findaid-idx?c=nlmfindaid;idno=nsmr> |
| **Institution** | Pro | Fund for the Replacement of Animals in Medical Experiments (FRAME) | FRAME promotes the elimination of the need to use laboratory animals through various activities including campaigning, publication of a scientific journal (ATLA), office-based and laboratory research and through its educational work ... FRAME’s ultimate aim is the elimination of the need to use laboratory animals in any kind of medical or scientific procedures. | <https://frame.org.uk/researching-alternatives-to-animal-testing/about-frame/> |
|  |  | The Jane Goodall Institute | The Jane Goodall Institute promotes understanding and protection of great apes and their habitat and builds on the legacy of Dr. Jane Goodall, our founder, to inspire individual action by young people of all ages to help animals... | <https://www.janegoodall.org/about/> |
|  |  | The Humane Society of the United States | We fight the big fights to end suffering for all animals. Together with millions of supporters, we take on puppy mills, factory farms, the fur trade, trophy hunting, animal cosmetics testing and other cruel industries. | <https://www.humanesociety.org/our-mission> |
|  |  | Center for the Expansion of Fundamental Rights (now called Nonhuman Rights Project) | To change the common law status of great apes, elephants, dolphins, and whales from mere “things,” which lack the capacity to possess any legal right, to “legal persons,” who possess such fundamental rights as bodily liberty and bodily integrity. | <https://www.nonhumanrights.org/who-we-are/> |
|  |  | New England Anti-Vivisection Society | We’re on a mission to save animals from suffering ... Giving you the facts about the worst animal abuses and how we’re ending animal testing once and for all. | <https://neavs.org/about-neavs> |
|  |  | Australian Association for Humane Research (now called Humane Research Australia) | Challenging the use of animal experiments and promoting more humane and scientifically-valid non-animal methods of research | <https://www.humaneresearch.org.au/> |
|  |  | In Defense of Animals | To rescue animals in need, foster respect for all sentient beings, and spark a revolution of compassion that liberates animals from the tyranny of systemic cruelty and exploitation. | <https://www.idausa.org/about-ida/> |
|  |  | Medical Advances Without Animals Trust (MAWA) | The aim of the Medical Advances Without Animals Trust (MAWA) is to advance medical science to improve human health and therapeutic outcomes without using animals or animal products. | [http://www.mawa-trust.org.au/#](http://www.mawa-trust.org.au/) |
|  |  | British Union for the Abolition of Vivisection | ...the British Union for the Abolition of Vivisection was an organisation created to campaign against vivisection of animals. | <http://www.hullhistorycentre.org.uk/research/research-guides/buav.aspx> |
|  |  | Americans For Medical Advancement | The organization opposes the use of animals as causal analogical models, or predictive models, for human response, and believes that using animals as a basis for the human response to drugs is not a safe method of development (Wikipedia, no active webpage) | <https://en.wikipedia.org/wiki/Americans_For_Medical_Advancement> |
|  |  | The Physicians Committee | Since 1985, the Physicians Committee has worked tirelessly for alternatives to the use of animals in medical education and research and advocating for more effective scientific methods... [Vision is C]reating a healthier world through a new emphasis on plant-based nutrition and scientific research conducted ethically, without using animals. | <https://www.pcrm.org/about-us> |
|  |  | Great Ape Project | The GAP Project – Great Ape Project defends the right of great apes to live in freedom in their habitats. From the moment they are deprived of this right and become victims of ill-treatment, lacking the ability to live in the forests, the mission is to offer the best quality of life and well-being possible to animals in the captivity regime. | <https://www.projetogap.org.br/en/mission-and-vision/> |
|  | Con | Federation of American Societies for Experimental Biology (FASEB) | The Federation of American Societies for Experimental Biology (FASEB) affirms the essential contribution of animals in research and education aimed at improving the health of both humans and animals. | <https://www.faseb.org/Science-Policy-and-Advocacy/Science-Policy-Research-Issues/Animals-in-Research-and-Education/Statement-of-Principles> |
|  |  | Southwest National Primate Research Center (SNPRC) | Our Mission is to improve the health of our global community through innovative biomedical research with nonhuman primates. | <https://snprc.org/about/> |
|  |  | Foundation for Biomedical Research (FBR) | The [FBR] ... is America’s most experienced, trusted and effective nonprofit dedicated to improving human and animal health by promoting public understanding and support for biomedical research. We believe that by illuminating the essential role animal research plays in changing health outcomes and defeating illnesses, we can help make lives even better. | <https://fbresearch.org/dedicated-to-animal-research/> |
|  |  | The Yerkes National Primate Research Center | Mission: To be recognized nationally and internationally as a leading center for both basic and applied research that involves our nonhuman primate colony and other animal species strategically in the service of humanity. | <http://www.yerkes.emory.edu/about/values.html> |
